# Abdominal wall and labial edema presenting in a girl with Henoch-Schönlein purpura: a case report

**DOI:** 10.1186/1752-1947-4-98

**Published:** 2010-03-29

**Authors:** Rania Hiram-Karasmanis, Ronald Garth Smith, Maria Radina, Donald Allen Soboleski

**Affiliations:** 1 Department of Pediatrics, Queen's University, Kingston, ON, Canada; 2 Faculty of Health Sciences, Queen's University, Kingston, ON, Canada; 3 Department of Diagnostic Radiology, Kingston General Hospital, Kingston, ON, Canada

## Abstract

**Introduction:**

Henoch-Schönlein purpura is a common immunoglobulin A-mediated vasculitic syndrome in children, characterized by purpuric rash, arthritis and abdominal pain. Renal involvement, manifested by the presence of hematuria and/or proteinuria, is also frequently seen. In most cases, patients with this disease achieve complete recovery, but some progress to renal impairment. Gastro-intestinal manifestations are present in two-thirds of affected patients and range from vomiting, diarrhea, and peri-umbilical pain to serious complications such as intussusception and gastrointestinal hemorrhage.

**Case presentation:**

We report the case of a 7-year-old Caucasian girl who presented with abdominal pain, labial swelling, and a large abdominal ecchymosis two weeks after having been diagnosed with Henoch-Schönlein purpura. A computed tomography scan revealed abdominal wall edema extending to the groin, without any intra-abdominal pathology. She was successfully treated with intravenous steroids.

**Conclusion:**

Circumferential anterior abdominal wall edema and labial edema have never been reported previously, to the best of our knowledge, as a complication of Henoch-Schönlein purpura. These findings further contribute to the wide spectrum of manifestations of this disorder in the literature, aiding in its recognition and management.

## Introduction

Henoch-Schönlein purpura (HSP) is an IgA-mediated vasculitis that presents with the common tetrad of abdominal pain, arthritis, purpuric rash and renal involvement. It is usually a benign disease of childhood, typically affecting children between the ages of four and seven years, who achieve complete recovery in most cases. HSP is often preceded by an upper respiratory tract infection, with Group A beta-hemolytic streptococcus responsible for up to 50% of the occurrences [1].

The diagnostic criteria of the American College of Rheumatology for HSP are the following: palpable purpura; patient is aged 20 years or above; acute abdominal pain; and biopsy showed granulocytes in the walls of small arterioles or venules [2].

The presence of two or more of these criteria has a sensitivity and specificity of 87.1% and 87.7%, respectively, for the diagnosis of HSP. A subsequent review of the classification of childhood vasculitides was carried out in 2005 and the diagnostic criteria modified (EULAR/PReS consensus report, 2006) [3]. These new criteria include:

Palpable purpura (mandatory criterion) in the presence of at least one of the following four features: Diffuse abdominal pain; any biopsy showing predominant IgA deposition; arthritis (acute, any joint) or arthralgia; renal involvement (any hematuria and/or proteinuria).

Gastrointestinal manifestations seen with HSP have been well described, and vary in their severity. In a study of 100 patients with HSP, Saulsbury reported that 63% of patients complained of abdominal pain [1] with typical symptoms including colicky abdominal pain, vomiting and gastrointestinal bleeding. Gastro-intestinal symptoms are the result of extravasation of blood and fluid into the bowel wall, leading to ulceration of the bowel mucosa and, eventually, bleeding, commonly involving the jejunum and ileum [4]. Intussusception is a rare but serious complication of HSP, occurring in 1-5% of patients [4]. In the past few years, there have been several case reports of new gastro-intestinal manifestations of HSP, including hemorrhagic ascites [5], perforation of large and small bowel [6], pancreatitis [7] and ischemic necrosis of the bile ducts [8].

Ultrasound is typically the modality of choice for investigation of abdominal pain associated with HSP, and can detect mural thickness and hematoma, ileus, peritoneal fluid and intussusception [9].

The treatment of HSP remains mainly supportive, as the acute symptoms resolve spontaneously in the majority of patients. For more severe cases with serious complications of the disease, treatment commonly includes corticosteroids, immunosuppressive drugs, and plasmapheresis [10]. The use of corticosteroids in the treatment of HSP remains anecdotal, as no prospective placebo controlled trials have been done [1]. A recent systematic review has been carried out by Weiss *et al*. in 2007 [11].

## Case presentation

A previously healthy 7-year-old Caucasian girl was admitted to our hospital with a history of abdominal pain, labial swelling and a large ecchymosis extending from the left subcostal area to the left lower quadrant. Two weeks before being admitted to the hospital, she experienced symptoms of an upper respiratory infection followed by joint discomfort, peripheral edema and a palpable, purpuric rash. She presented to a smaller community hospital, where the additional findings of hypertension and a Group A beta-hemolytic streptococcus-positive throat swab were discovered by a consulting pediatrician. She was diagnosed with Henoch-Schönlein purpura (HSP) and treated with two weeks of Penicillin V for her tonsillitis. Her symptoms improved, but over the course of the next six days, she developed increasing abdominal pain and "distention". She also experienced significant pain in her genital area with associated labial swelling. She was transferred to our institution, a tertiary care Pediatric Center, for further evaluation. There was no history of abdominal trauma.

**Figure 1:**
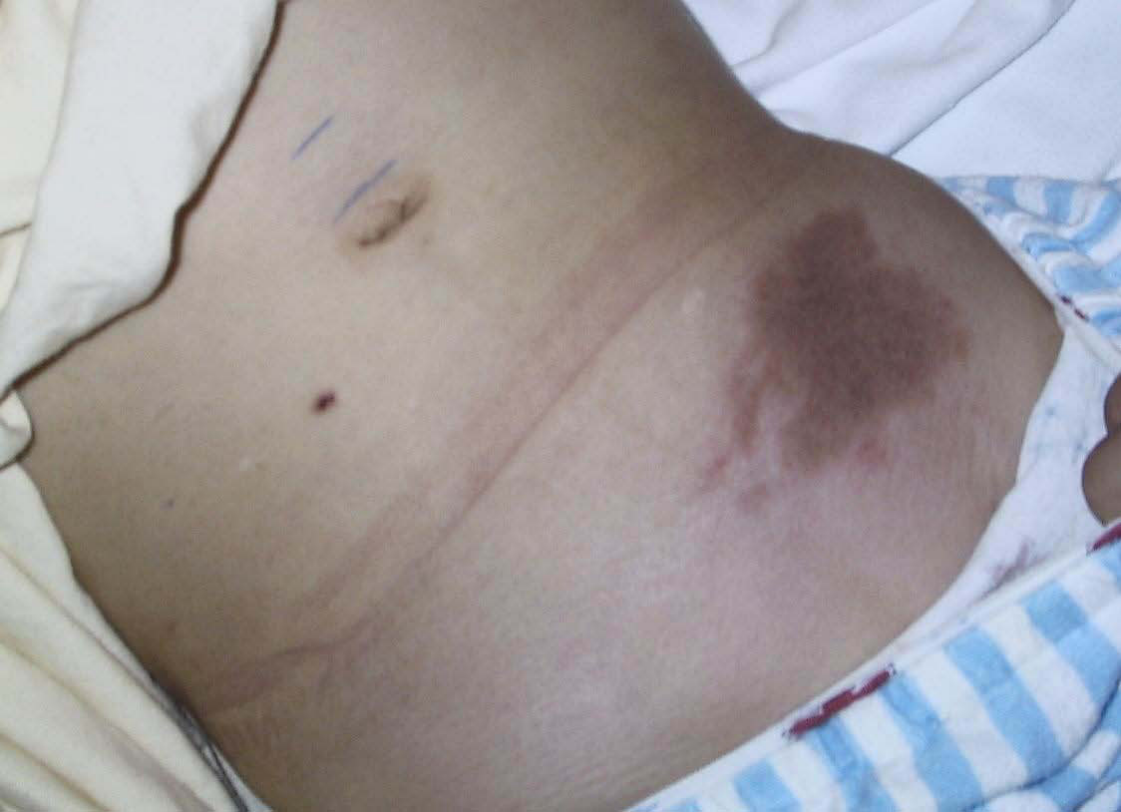
**Abdominal ecchymosis**. Large ecchymosis over the left lower quadrant and edema of the left flank.

**Figure 2:**
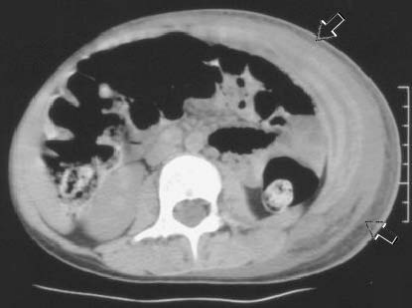
**Abdominal wall edema**. Unenhanced axial computed tomography scan through the mid-abdomen demonstrates extensive fluid tracking between abdominal wall muscle layers and coursing along anterior margins within subcutaneous fat (arrows).

**Figure 3:**
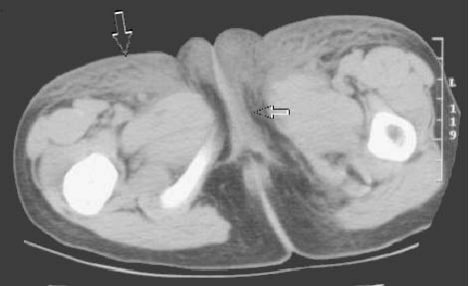
**Labial edema**. Unenhanced axial computed tomography image depicts extensive bilateral subcutaneous edema (large arrow) with extension into the labia (small arrow).

On admission, she was found to be hypertensive, with a blood pressure just above the 90th percentile for age and height. She had an exudative plaque on the left tonsil, but a throat swab was negative. Her abdomen revealed a large ecchymosis, 10 cm in diameter over the left quadrant, with significant edema/swelling extending from the left flank to the labia majora (Figure 1). The abdomen was diffusely tender to palpation, and a faint raised papular rash was noted on her lower abdomen, buttocks and lower extremities. A complete blood count revealed a leukocytosis with a left shift and a normal platelet count. Urine analysis was positive for blood (microscopic) and an abdominal ultrasound was "normal" but incomplete. The ultrasound was performed to rule out intussusception, but had to be abandoned due to the extreme abdominal wall tenderness. A computed tomography (CT) scan of the abdomen was then performed, and this revealed markedly increased attenuation within the subcutaneous tissues of the left side of the abdominal wall and flank, extending to the groin and labia majora, consistent with hemorrhage or edema (Figure 2, 3). No intra-abdominal pathology was seen. Our patient was treated with 2 mg/kg/day of intravenous methylprednisolone for 48 hours, and then switched to 2 mg/kg/day of oral prednisone for seven days. Within 36 hours of initiation of treatment, her abdominal pain and distention improved significantly and her hematuria resolved.

## Discussion

The reported case describes circumferential anterior abdominal wall edema and labial edema as a complication of HSP. Gastrointestinal manifestations of HSP are common. However, they usually result from edema and ulceration of the bowel wall, leading to pain and hemorrhage. Although only a minority of patients with HSP are investigated with a CT scan, findings usually include multifocal bowel wall thickening with skip areas, mesenteric edema, vascular engorgement and non-specific lymphadenopathy [12]. Our patient had a normal bowel wall, but instead had significant circumferential edema within the abdominal wall and the left flank -- a finding that has not been previously reported. A CT scan was performed mainly because of the acute tenderness of the abdominal wall, which precluded satisfactory completion of the ultrasound study.

Genital involvement in HSP has been previously documented in males, and consisted of orchitis and scrotal swelling found in nine percent of boys in Saulsbury's study [1]. There were also two recent reports of penile edema associated with HSP [13,14]. Aside from one report of cutaneous vulvar lesions associated with HSP, there have been no other descriptions of involvement of the women's genitalia to date [15]. Our patient had clinical and radiological edema of the labia majora, which was likely an extension of the abdominal wall edema. A finding such as this has never been reported before, and may be the female equivalent of scrotal swelling in boys.

Similar to the responses described in several other reports, our patient had significant improvement within hours of initiation of steroid treatment and achieved almost complete resolution of symptoms within three days.

## Conclusion

This case presents an occurrence of abdominal wall and labial edema as a complication associated with HSP that has not been previously reported. The symptoms resolved promptly upon treatment with steroids. These new findings are of interest, as they further contribute to the wide spectrum of manifestations of HSP already described in the literature, aiding in the recognition and management of this disorder.

## Abbreviations

CT: Computed tomography; HSP: Henoch-Schönlein purpura; kg: Kilograms; mg: Milligrams.

## Consent

Written informed consent was obtained from the parent of our patient for publication of this case report and any accompanying images. A copy of the written consent is available for review by the Editor-in-Chief of this journal.

## Competing interests

The authors declare that they have no competing interests.

## Authors' contributions

RH and RGS conceived of the project and participated in its design. MR and RH helped draft the manuscript. DS assisted with the interpretation of radiological data. All authors read and approved the final manuscript.

## References

[B1] Saulsbury FT Henoch-Schönlein purpura in children. Report of 100 patients and review of the literature Medicine 1999 78 395 409 10.1097/00005792-199911000-00005 10575422

[B2] Mills JA Michel BA Bloch DA Calabrese LH Hunder GG Arend WP Edworthy SM Fauci AS Leavitt RY Lie JT The American College of Rheumatology 1990 criteria for the classification of Henoch-Schönlein purpura Arthritis & Rheumatism 1990 33 1114 1121 10.1002/art.1780330809 2202310

[B3] Ozen S Ruperto N Dillon MJ Bagga A Barron K Davin JC Kawasaki T Lindsley C Petty RE Prieur AM Ravelli A Woo P EULAR/PReS e ndorsed consensus criteria for the classification of childhood vasculitides Annals of the Rheumatic Diseases 2006 65 936 941 10.1136/ard.2005.046300 16322081 PMC1798210

[B4] Bailey M Chapin W Licht H Reynolds JC The effects of vasculitis on the gastrointestinal tract and liver Gastroenterology Clinics of North America 1998 27 747 782 10.1016/S0889-8553(05)70032-7 9890113

[B5] Venuta A Bertolani P Garetti E Venturelli C Predieri B Muttini ED Compagni E Hemorrhagic ascites in a child with Henoch-Schönlein purpura Journal of Pediatric Gastroenterology & Nutrition 1999 29 358 359 10.1097/00005176-199909000-00023 10468007

[B6] Bissonnette R Dansereau A D'Amico P Pateneaude JV Paradis J Perforation of large and small bowel in Henoch-Schönlein purpura International Journal of Dermatology 1997 36 361 363 9199985 10.1111/j.1365-4362.1997.tb03098.x

[B7] Cheung KM Mok F Lam P Chan KH Pancreatitis associated with Henoch-Schönlein purpura Journal of Paediatrics & Child Health 2001 37 311 313 10.1046/j.1440-1754.2001.00606.x 11468053

[B8] Viola S Meyer M Fabre M Tounian P Goddon R Dechelotte P Valayer J Gruner M Bernard O Ischemic necrosis of bile ducts complicating Schönlein-Henoch purpura Gastroenterology 1999 117 211 214 10.1016/S0016-5085(99)70569-X 10381929

[B9] Connolly B O'Halpin D Sonographic evaluation of the abdomen in Henoch-Schönlein purpura Clinical Radiology 1994 49 320 323 10.1016/S0009-9260(05)81796-9 8013195

[B10] Roberts PF Waller TA Brinker TM Riffe IZ Sayre JW Bratton RL Henoch-Schonlein purpura: a review article Southern Medical Journal 2007 100 821 824 17713309 10.1097/SMJ.0b013e3180f62d0f

[B11] Weiss PF Feinstein JA Luan X Burnham JM Feudtner C Effects of corticosteroid on Henoch-Schönlein purpura: a systematic review Pediatrics 2007 120 1079 1087 10.1542/peds.2007-0667 17974746 PMC3525094

[B12] Jeong YK Ha HK Yoon CH Gong G Kim PN Lee MG Min YI Auh YH Gastrointestinal involvement in Henoch-Schönlein syndrome: CT findings AJR American Journal of Roentgenology 1997 168 965 968 9124151 10.2214/ajr.168.4.9124151

[B13] Sandell J Ramanan R Shah D Penile involvement in Henoch-Schönlein purpura Indian Journal of Pediatrics 2002 69 529 530 10.1007/BF02722659 12139142

[B14] Pennesi M Biasotto E Saccari A Schönlein-Henoch purpura involving the penis Archives of Disease in Childhood 2006 91 603 10.1136/adc.2005.072652 16790720 PMC2082837

[B15] Fischer G Rogers M Vulvar disease in children: a clinical audit of 130 cases Pediatric Dermatology 2000 17 1 6 10.1046/j.1525-1470.2000.01701.x 10720979

